# A precursor mechanism triggering the second magnetization peak phenomenon in superconducting materials

**DOI:** 10.1038/s41598-021-86728-8

**Published:** 2021-03-31

**Authors:** M. Polichetti, A. Galluzzi, K. Buchkov, V. Tomov, E. Nazarova, A. Leo, G. Grimaldi, S. Pace

**Affiliations:** 1 Department of Physics “E.R. Caianiello”, University of Salerno, Via Giovanni Paolo II, 132, 84084 Fisciano, Salerno Italy; 2 CNR-SPIN Salerno, Via Giovanni Paolo II, 132, 84084 Fisciano, Salerno Italy; 3 Institute of Solid State Physics, Bulgarian Academy of Sciences, 72 Tzarigradsko Chaussee, 1784 Sofia, Bulgaria; 4 Institute of Optical Materials and Technologies, Bulgarian Academy of Sciences, Acad. G. Bonchev Str. Bl. 109, 1113 Sofia, Bulgaria

**Keywords:** Superconducting properties and materials, Condensed-matter physics, Magnetic properties and materials

## Abstract

The correlation in type-II superconductors between the creep rate S and the Second Magnetization Peak (SMP) phenomenon which produces an increase in J_c_, as a function of the field (H), has been investigated at different temperatures by starting from the minimum in S(H) and the onset of the SMP phenomenon detected on a FeSe_0.5_Te_0.5_ sample. Then the analysis has been extended by considering the entire S(H) curves and comparing our results with those of many other superconducting materials reported in literature. In this way, we find evidence that the flux dynamic mechanisms behind the appearance of the SMP phenomenon in J_c_(H) are activated at fields well below those where the critical current starts effectively to increase. Moreover, the found universal relation between the minimum in the S(H) and the SMP phenomenon in J_c_(H) shows that both can be attributed to a sequential crossover between a less effective pinning (losing its effectiveness at low fields) to a more effective pinning (still acting at high fields), regardless of the type-II superconductor taken into consideration.

## Introduction

An important parameter that takes into account the vortex activity inside a type-II superconductor is the normalized relaxation rate or simply the creep rate (S) that can be obtained by time dependent magnetic measurements^1^. Generally, an increase of the creep rate is associated to a suppression of the critical current density of the material, and the comprehension of the mechanisms underneath its evolution with temperature and magnetic field can help to improve the electrical transport properties of the superconductors. Based on the typology of the superconductor, the creep rate reveals different field and temperature dependencies. In Low Temperature Superconductors (LTS) a linear increase of the creep rate with the temperature is detected and predicted by the Kim-Anderson model^2,3^. The High Temperature Superconductors (HTS) more complex vortex matter properties result in a strong vortex motion and fluctuations^4^, due to their larger anisotropy, shorter coherence lengths and higher critical temperatures. For this class of materials, a plateau in the temperature dependence of the creep rate S(T) has been observed^1,5–9^. Such an anomalous behavior with respect to the LTS has been interpreted in the framework of the collective creep theory^1,4,6^ that considers significant interaction among the vortices inside the material at high fields. Relaxation measurements performed on Iron Based Superconductors (IBS) have shown high creep rate values and the presence of the plateau in the S(T) curve in agreement with the HTS^10–17^. Very often the region defined as the plateau in the S(T) behavior is characterized by the presence of a minimum^14,16,18,19^, also visible in the field dependence of the creep rate S(H)^16,18–20^. Independently of the class of the superconductor, these creep rate characteristics have been often reported in concomitance with the presence of the second magnetization peak (or just simply “peak effect”) phenomenon^17,18,20–28^. It is necessary to consider that in literature it is common to find both the notations “Second Magnetization Peak”^15,22,29^ and “Peak Effect”^18,30–32^ for this kind of phenomena where the peak in the field dependence of the critical current density occurs away from H_c__2_. Here, we use the notation Second Magnetization Peak (SMP). The SMP phenomenon manifests as a particular shape of the magnetic hysteresis loop due to an anomalous modulation of the critical current density with increasing field. Within the SMP phenomenon, the physical mechanism which is responsible for the presence of the “second peak” (that is the maximum in the magnetization and therefore in the critical current) has been extensively studied and understood, and it is widely accepted that the maximum in J_c_ has to be related to an “elastic to plastic crossover” ^13,14,16,17,22,30,33–36^. It is also worth to underline that the regime between the onset point and the maximum point of the SMP phenomenon has been ascribed to (i) a more-or-less formal phase transition of vortex matter from an ordered state to a disordered one (Bragg glass to amorphous glass), (ii) a crossover from an elastic to a plastic deformational behavior of vortex matter, (iii) to a three-dimensional to two-dimensional crossover (or formal transition) etc^37–43^. It is important to underline that in this work we will focus on the precursor mechanism that leads to the beginning of the SMP phenomenon (onset), and so at fields below the onset field. Nevertheless, the presence of both the plateau and the minimum in the S(T,H) curve has not been completely correlated to the SMP phenomenon, although some efforts in this direction have been made looking for a correlation between the minimum in the S(T,H) curve and the second peak^13,14,16,22^. In this paper no particular discussion has been done about the relation between the flux dynamics and the second peak since it has been extensively studied and understood. In particular, a complete phase diagram of vortex matter has been drawn for our sample (see main panel of Fig. 1 in Supplementary Information and Ref.^44^ for details) together with the H_sp_(T) curve behavior close to T_c_ (see inset of Fig. 1 in Supplementary Information and Ref.^44^ for details).On the other hand, until now just a little attention has been given to the possibility that a correlation between the minimum of the creep rate and the onset of the SMP phenomenon could exist^45,46^. The term “onset” indicates where the J_c_ inverts its decreasing behavior and starts to increase with field. In general, the triggering cause of the SMP phenomenon is still in the research focus since this phenomenon seems to be due to a different mechanism depending on the analyzed sample. Finding a unique interpretation of the SMP phenomenon trigger which produces an increasing J_c_(H) behavior would be desirable and useful for its complete interpretation, due to its prospective exploitation of superconductivity for power applications.

**Figure 1:**
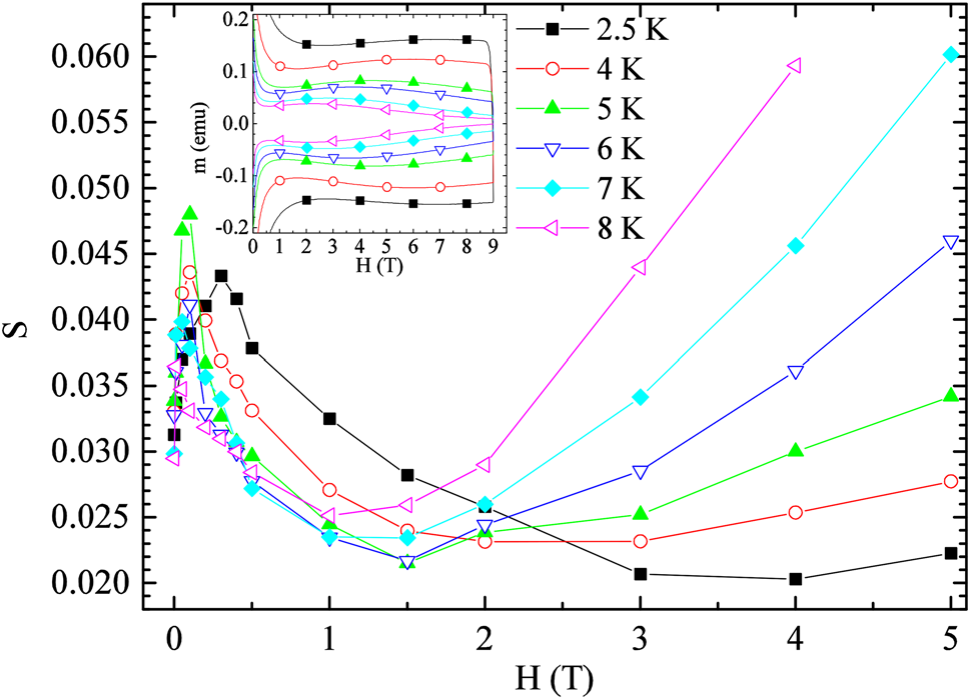
Field dependence of the normalized relaxation rate S. The solid lines are only a guide for the eyes. Inset: The SMP phenomenon in the superconducting hysteresis loops is shown.

For this purpose the paper attention is focused on the correlation between the onset of the SMP phenomenon and the minimum of the creep rate by starting from the magnetic relaxation of a FeSe_0.5_Te_0.5_ crystal, in which a SMP phenomenon through dc magnetization measurements is detected. From the relaxation measurements, the temperature (T) and the field (H) dependence of the normalized relaxation rate (S) have been obtained. The S(T,H) curves show high values and a minimum, as already reported for HTS and other IBS, and we present a detailed analysis of S(T,H) behavior, extended to a large set of other superconducting materials, which probes the existence of a correlation between the SMP phenomenon and the creep rate.

## Results and Discussion

In order to study the characteristics of relaxation phenomena acting in our sample that presents a SMP phenomenon (see inset Fig. 1), measurements of magnetization as a function of time have been performed at different temperatures from 2.5 K up to 8 K and magnetic fields between 0.01 T and 7 T. Starting from these measurements, the field dependence of the normalized creep rate $S=\left|dlnM/dlnt\right|$ has been obtained^1^, where *M* is the magnetization normalized by its first value and *t* is the time measured from the moment the field reaches the target value. The field dependence of *S* is shown in Fig. 1. For all the reported temperatures it is visible an initial increase of the creep rate values with increasing field followed by a decrease that precedes a further increase for higher fields. It is worth to underline the presence of a minimum in the S(H) curves.

**Figure 2:**
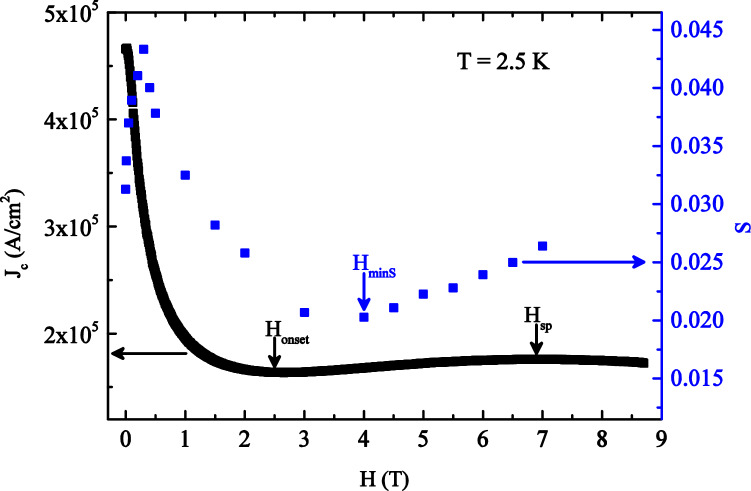
The field dependence of relaxation rate S (blue closed squares, right scale) shown together with the J_c_(H) curve (black closed squares, left scale) measured at the same temperature T = 2.5 K.

The minimum in the S(H) curves is associated with the lowest creep activity of vortices inside the sample and it has been already observed along with the presence of the SMP phenomenon^17,18,20,22,47^, although their correlation still presents a puzzling challenge. For this purpose, the S(H) curve at T = 2.5 K has been plotted together with the J_c_(H) curve at the same temperature in order to search for correlation between the creep rate and the SMP phenomenon. The J_c_(H) curve has been calculated using the Bean critical state model (see Supplementary Information for additional details). As shown in Fig. 2, the minimum in the S(H) curve (H_minS_) is placed between the onset of the SMP phenomenon (H_onset_) and the second peak position (H_sp_). This feature has been found for all the investigated temperatures. Moreover, we already checked^48^ that the variation of the sweep rate influences in a negligible way the onset field position H_onset_ (between 0.1% and 7%).

Comparing the behavior reported in Fig. 2 with analogous results reported in the literature, we note the same correlation in the position of H_onset_, H_minS_ and H_sp_. In particular, the ratio R between H_onset_ and H_minS_ values reported in Fig. 2, is R ≈ 0.6 (at T = 2.5 K). Coherently, the data reported in literature show that this ratio is always lower than 1 for several type II superconductors, as reported in Table 1 for different materials (*Sample*) at a given measurement temperature (*T(K)*).

**Table 1:** Ratio H_onset_ /H_minS_ (R) for several type II superconductors.

Sample	T (K)	R(H_onset_ /H_minS_)	References
YBa_2_Cu_3_O_7-δ_	77	0.51 ± 0.05	Küpfer et al.^49^
La_1.88_Sr_0.12_CuO_4_	16	0.40 ± 0.04	Ionescu et al.^50^
La_1.8_Sr_0.2_CuO_4_	26	0.28 ± 0.03	Ionescu et al.^50^
Bi_2_Sr_2_CaCu_2_O_8+δ_	25	0.74 ± 0.04	Miu et al.^23^
YBa_2_CuO_7-δ_	40	0.22 ± 0.02	Pissas et al.^24^
(K, Ba)BiO_3_	4	0.67 ± 0.03	Joumard et al.^51^
Ba(Fe_0.93_Co_0.07_)_2_As_2_	15	0.30 ± 0.03	Prozorov et al.^16^
FeTe_0.6_Se_0.4_	5	0.54 ± 0.06	Sun et al.^20^
Ca_10_(Pt_4_As_8_)(Fe_1.99_Pt_0.01_As_2_)_5_	12	0.45 ± 0.05	Ahmad et al.^52^
FeTe_0.7_Se_0.3_	4.25	0.57 ± 0.05	Bonura et al.^18^
BaFe_2_(As_0.72_P_0.28_)_2_	18	0.70 ± 0.03	Ionescu et al.^50^
Ba(Fe_0.93_Co_0.07_)_2_As_2_	15	0.40 ± 0.03	Nakajima et al.^53^

More interestingly, the value of the ratio R = 0.6 ± 0.04 in our sample stays approximately constant (within 7%) at all the investigated temperatures (in the range of T/T_c_ between 0.17 and 0.55). For T > 0.55T_c_ the ratio cannot be calculated since the minimum of creep rate moves with temperature to a value of field which cannot be experimentally evaluated. The temperature independence of R can be found in other superconducting materials by using the data extracted from the literature, as indicated in Table 2 for an additional list of materials (*Sample*) at various measurement temperatures (*T(K)*). This suggests a sort of universal correlation between the minimum of the creep rate and the beginning of the dynamic phenomena that trigger the increase of the critical current (onset), terminating with a peak in J_c_(H) (second peak), which is not depending on the sample. It must be pointed out that the different anisotropy values of the samples can affect the effectiveness of the pinning typologies inside the sample^21,43^. In fact, the trends of the curves are the same for the materials with different anisotropies but the fields where the different non-monotonicities of the curves appear are different, as it is plausible to expect for materials with different characteristics including anisotropy. Nevertheless, the temperature independence of R is verified for superconductors with low, intermediate, and high anisotropy values as reported in Table 2. On the other hand, the ratio between the second peak position and the minimum in the S(H) curve (H_sp_/H_minS_) is not constant. The different ratio behavior as a function of temperature between H_onset_ /H_minS_ and H_sp_/H_minS_ confirms that the phenomena associated to the appearance of the onset and the second peak are different.

**Table 2:** Ratio H_onset_ /H_minS_ (R) for several type II superconductors at various temperatures.

Sample	T (K)	R(H_onset_ /H_minS_)	References
Bi_2_Sr_2_CaCu_2_O_8+δ_	16, 25	0.37 ± 0.02	Sun et al.^25^
LiFeAs	5, 10	0.67 ± 0.05	Pramanik et al.^14^
HgBa_2_CuO_4+δ_	5, 7.5, 10	0.69 ± 0.05	Pissas et al.^26^
FeTe_0.6_Se_0.4_	9, 10, 11	0.47 ± 0.05	Ionescu et al.^50^; Miu et al.^54^
Ba_0.75_K_0.25_Fe_2_As_2_	9, 11 12	0.61 ± 0.03	Sundar et al.^55^
Ba(Fe_0.935_Co_0.065_)_2_As_2_	15, 16, 19	0.32 ± 0.02	Sundar et al.^29^
Ca_0.8_La_0.2_Fe_0.978_Co_0.022_As_2_	16, 20, 25	0.50 ± 0.03	Zhou et al.^15^
FeTe_0.59_Se_0.41_	2, 4, 6	0.45 ± 0.05	Taen et al.^19^
Ba_0.72_K_0.28_Fe_2_As_2_	25.5, 27.2, 29.5	0.26 ± 0.03	Salem-Sugui et al.^22^
Nb	2.5, 3.5, 4.3, 5	0.69 ± 0.07	Stamopoulos et al.^27^
BaFe_2_(As_0.68_P_0.32_)_2_	21, 22, 23, 24	0.43 ± 0.05	Salem-Sugui et al.^56^
Ba(Fe_0.925_Co_0.075_)_2_As_2_	12, 14, 18, 19	0.31 ± 0.04	Kopeliansky et al.^17^
Fe_0.96_Te_0.59_Se_0.45_	2.5, 4, 5, 6, 7, 8	0.6 ± 0.04	This work

**Figure 3:**
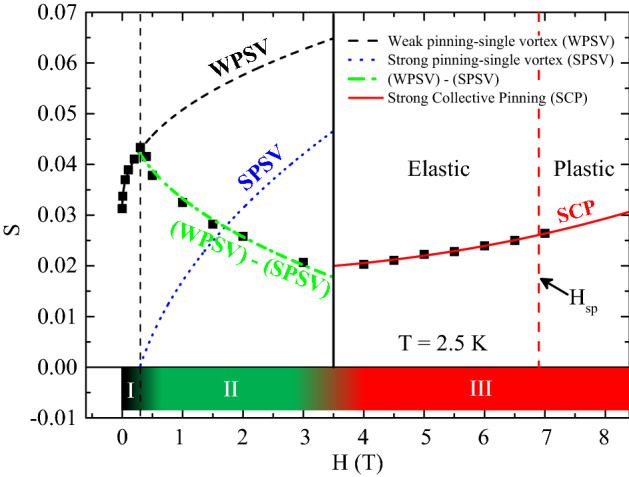
S as a function of H at T = 2.5 K. The black dashed line is the fit of the first S(H) increase with Eq. (1). The blue dotted line is the strong pinning single vortex behavior speculated for the vortices that enter in the twin boundaries using Eq. (1). The green dashed-dotted line is the fit of the decreasing S(H) data with the equation described by the subtraction of the black and blue line. The red solid line is the fit of the second S(H) increase with Eq. (1). In the bottom of the figure, the field intervals relative to the three S(H) portions are identified with different colours. Finally, the black vertical solid line separates the single vortex state from the collective pinning state while the red vertical dashed line, individuated by the H_sp_ value, separates the elastic regime from the plastic regime in the framework of the collective pinning theory.

The constancy of R with temperature suggests that both the onset of the SMP phenomenon and the minimum of the creep rate, with field, are different aspects of the same basic phenomenon. Therefore, since in the analyzed sample the SMP phenomenon has been correlated to a field driven crossover^44^ from a less effective type of pinning due to point-like defects, to a more effective one due to twin boundaries^57^, we have analyzed the S(H) behavior by assuming the same mechanism. In particular, due to the relation $S\propto {U}^{-1}$ between the creep rate and pinning energy U (with U ranging between 75 K at T = 2.5 K and 325 K at T = 9.5 K in our material^36^) and since the literature^4,58,59^ suggests to analyze the field dependence of the pinning energy by the relation $U(H)\propto {H}^{-\alpha }$, the value of α, which indicates the pinning regime acting in the sample, has been extracted from the S(H) curves by using the equation.

1 $$S(H) = S(0) + bH^{\alpha}.$$

where S(0) is the value of S at H = 0 T and b is a constant. The possible influence in the S(H) data of an eventual surface barrier has been excluded in our preliminary analysis of the data, since the characteristic fingerprints of its presence has been revealed neither in the m(H) curves^60–62^ (see inset of Fig. 1), nor in the relaxation measurements^63^ (see Fig. 3 of Supplementary Information file). Looking at Fig. 3, three portions of the S(H) experimental curve can be individuated: (I) S increases for low fields (0 T ≤ H ≤ 0.3 T); (II) S decreases for intermediate fields (0.3 T < H $\lesssim$ 3 T); (III) S increases again for high fields (H ≥ 4 T). These three portions can therefore be analyzed separately by using Eq. (1). From the fit of the data in portion (I) the value of α = 0.434 has been obtained, so suggesting that the magnetic behavior is due to a single vortex state^4,58,64^ with the vortices that start to penetrate the material in the point-like defects without interacting with each other in this considered field range^65^. On the other hand, in the portion (III) the analysis returns a value of the exponent α = 2.395 which corresponds to a strong collective pinning regime^4,58,64^, in presence of the same crossover observed in Krusin-Elbaum et al. paper^66^ and also as expected due to both the high values of the applied field and the presence of strong pinning coming from the twin boundaries detected in our sample. Consequently, the portion (II) at intermediate fields can be interpreted in terms of vortices leaving the weak defects and accommodating in the strong defects, as also reported by Mohan et al. paper^67^, while new other vortices enter in the weak defects with increasing field, still in single vortex state. This accommodation in the strong defects can be represented in Fig. 3 by a curve that has to start from 0 at H = 0.3 T, and to have the same exponent α = 0.434 as the one already found for the single vortex state. This curve is represented by the blue dotted line in Fig. 3. So, the decreasing S(H) data can be fit with an expression composed by the subtraction of the equation describing the black dashed line (weak single vortex) with the one describing the blue dotted line (strong single vortex) of Fig. 3. In this way, we obtain the green dashed-dotted curve which well represents our data in the intermediate field region where all the vortices are expected to be in a single vortex regime^4,58,64^. It is worth to underline that even though 2–3 T could appear a high field for considering single vortex state, there are several works in literature which explain their results in terms of single vortex state also at elevate magnetic fields such as 2–3 T^58,64,68–70^. The described mechanism has been verified also for other temperatures, where it was still possible to detect the presence of a portion (I) in the S(H) data. So, in this scenario, for increasing field, the creep rate, after the initial growth due to a less effective pinning, starts to decrease because of a crossover from a weak to a stronger pinning reaching a minimum after which it increases by following the dynamics regulated by the more effective pinning centers. Therefore, the weak to strong pinning crossover, responsible for the onset of the SMP phenomenon, starts where the S(H) curve reaches its maximum at low fields, and this happens at fields lower than the detectable beginning of the SMP phenomenon which is identified at H_onset_.

**Figure 4:**
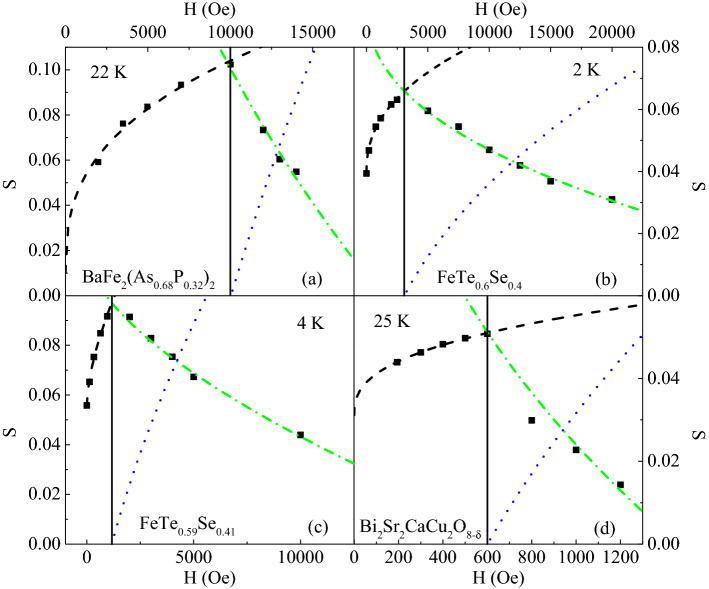
Fit procedure of Fig. 3 applied to the S as a function of H curves of different samples. Data have been taken from literature, in particular from^56^ (for the panel (a)),^20^ (for the panel (b)),^19^ (for the panel (c)), and^25^ (for the panel (d)).

The discussed analysis has been tested also on some experimental data of three different iron based and one cuprate superconductors available in literature^19,20,25,56^, since in those cases all the necessary S(H) values (at low, intermediate and high fields) for the analysis were reported. The data have been extracted from the plots reported in the papers by means of a specific digitization software^71^ and the same kind of fits, discussed in this work, have been applied (see Fig. 4). The agreement is excellent for the IBS materials, although with slightly different α values respect to our sample, whereas for the cuprate superconductor the agreement is still good although less satisfactory due to the low number of available experimental points.

## Conclusions

The magnetic relaxation phenomena of a FeSe_0.5_Te_0.5_ crystal displaying a SMP phenomenon have been analyzed by means of dc magnetization measurements as a function of time. From the experimental data, we have calculated the creep rate S as a function of the magnetic field H at different temperatures, which shows the presence of a minimum in the S(H) curves. This minimum is visible in a large number of works present in literature, where it is possible to note also the presence of a SMP phenomenon in the critical current density J_c_(H). This is the first indication that the two phenomena are correlated. In particular, we have observed that the onset of the SMP phenomenon (H_onset_) always occurs at fields lower than the S(H) minimum (H_minS_) and that the ratio R between H_onset_ and H_minS_ is temperature independent, although assuming different values for the different materials analyzed. As the creep rate S is expected to grow up when the magnetic field starts to increase from zero, due to the natural reduction of the pinning energy with field, it follows that the existence of a minimum at a given field in the S(H) curve must suggest that a maximum at lower fields is present. This is clearly pointed out in our data and is also observed in other works in literature where measurements of S(H) at low enough fields are performed and reported. Since in a previous work the correlation between the SMP phenomenon and the crossover from a weak to a stronger pinning was evidenced, and due to the temperature independent value of the ratio R which relates the behavior of the SMP phenomenon to that of the creep rate S(H), it appears plausible to also analyze the S(H) curves in terms of a pinning crossover. This approach, indeed, results in an excellent agreement with the crossover hypothesis, and so indicates also that the flux dynamic mechanisms producing the SMP phenomenon are activated at fields well below the onset. The discussed analysis has been applied also to some other experimental data available in literature, and the agreement is very satisfactory. The strong analogy between the data experimentally obtained from our material and those present in literature would suggest that also in the other cases the phenomenon of the SMP, and the correlated one of the minimum in the S(H) curve, can be preceded and triggered by a sequential crossover between a less effective pinning (at low fields) to a more effective pinning (at high fields). Such a pinning crossover occurs in the region of the field that precedes the appearance of the elastic to plastic transition corresponding to the maximum in J_c_. This universal behavior appears to be independent of the particular type of pinning centers involved in the phenomenon, the presence of specific transitions in the vortex lattice that make the same pinning centers more effective and the structure of the material or of its belonging to the family of low or high-T_c_ superconductors as well.

## Methods

We have analyzed a FeSe_0.5_Te_0.5_ (nominal composition) crystal with dimensions 3 × 3 × 0.2 mm^3^, produced by means of Bridgman technique and having T_c_ = 14.5 K. Fabrication details are reported elsewhere^44^. A SEM–EDX analysis performed on the sample has shown the presence of twin boundaries and a slight deviation of the stoichiometry (Fe_0.96_Te_0.59_Se_0.45_) from the nominal composition^72^. This is probably due to micro inhomogeneity and phase separation of magnetic premises, typical for the crystal growth and synthesis in FeSeTe^73–76^ and its basic compound FeSe^77,78^. The sample has been characterized in a dc magnetic field applied perpendicular to its largest face. In particular, the dc magnetization as a function of the time M(t) has been measured using a Quantum Design PPMS-9 T equipped with a VSM option. To avoid the effect on the sample response due to the residual trapped field inside the PPMS dc magnet**,** this field was reduced below 1 × 10^–4^ T following the procedure described in a previous paper^79^.

To measure the relaxation of magnetization as a function of time M(t), the temperature of the sample was first stabilized to the measurement value in the absence of a field. After that, 8 T magnetic field was applied and then reduced to the target field with a sweep rate of 100 Oe/s following the procedure indicated in Ref.^1^. After waiting 100 s, the magnetization data was acquired for a period of 10,800 s. Raw data of M(time) are shown in Ref.^36^ (see also Supplementary Information for additional details).

## Supplementary Information

Supplementary Information

## Data Availability

The data sets that support the findings in this study are available from the corresponding author upon reasonable request.
