# Supplementary material for: A precursor mechanism triggering the second magnetization peak phenomenon in superconducting materials
Source: Sci Rep. 2021 Mar 31;11:7247. doi: 10.1038/s41598-021-86728-8 (PMC8012359; doi:10.1038/s41598-021-86728-8)
Supplement: Supplementary file 1 — Supplementary Information [file 41598_2021_86728_MOESM1_ESM.pdf]

[Supplementary Information] A precursor mechanism triggering the second magnetization peak phenomenon in superconducting materials

M. Polichetti[1,2,*,+], A. Galluzzi[1,2,+], K. Buchkov[3,4], V. Tomov[3], E. Nazarova[3], A. Leo[1,2], G. Grimaldi[2], S. Pace[1,2]

[1]Department of Physics “E.R. Caianiello”, University of Salerno, via Giovanni Paolo II, 132, Fisciano (SALERNO), I-84084, Italy

[2]CNR-SPIN Salerno, via Giovanni Paolo II, 132, Fisciano (SALERNO), I-84084, Italy

[3]Institute of Solid State Physics, Bulgarian Academy of Sciences, 72 Tzarigradsko Chaussee, 1784 Sofia, Bulgaria

[4]Institute of Optical Materials and Technologies, Bulgarian Academy of Sciences, Acad. G. Bonchev Str. Bl. 109, Sofia, 1113, Bulgaria

*corresponding author: polimax@sa.infn.it
+these authors contributed equally to this work

Vortex phase diagram H(T)

The complete phase diagram of vortex matter for our sample in the entire H-T regime accessible by our experiments has been reported in Figure 1. In the main panel of Figure 1, the H(T) region regarding the precursor mechanisms that lead to the birth of the Second Magnetization Peak (SMP) phenomenon, which are the subject of our study in this work, has been indicated by an arrow. It is worth to underline how this field-temperature region is narrow and located before H_{onset}, a rarely explored region so far. It can be also important to specify that H_{sp} curve is far from H_{c2} curve indicating that, formally, we are considering a SMP phenomenon. It is necessary to consider that in literature it is common to find both the notations “Second Magnetization Peak”[1–3] and “Peak Effect”[4–7] for this kind of phenomena where the peak in the field dependence of the critical current density occurs away from Hc_2. Here, we use the notation Second Magnetization Peak. In the inset of Figure 1, an enlargement of the region near T_c has been reported. It can be noted that the H_{sp} line (red solid line) terminates at 12.5 K and does not join the H_{irr} and H_{c2} line due to a possible reordering of vortex matter near the end point of the second peak line as reported in Refs.[8,9].

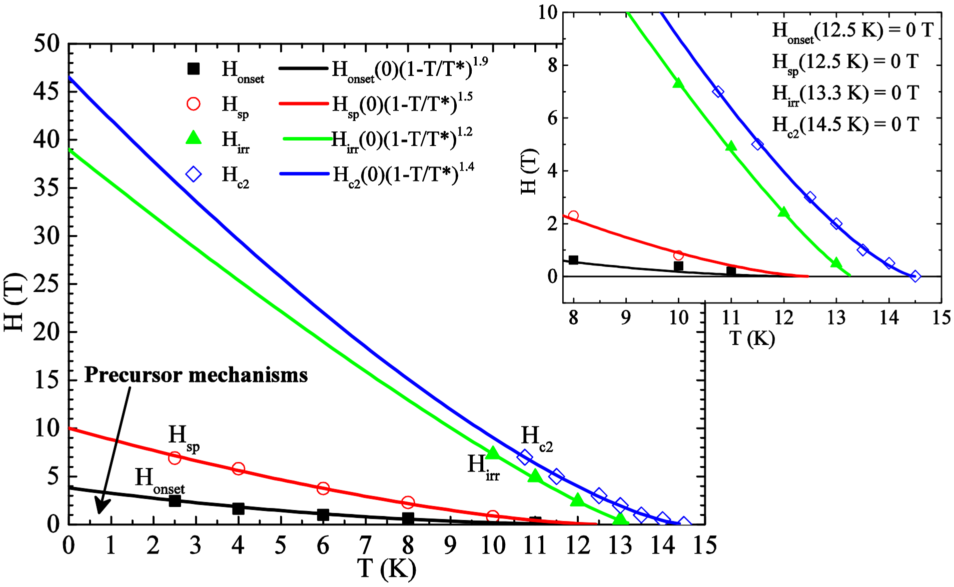

Figure 1 The field-temperature vortex phase diagram of $FeSe_{0.5}Te_{0.5}$ crystal. The characteristic field H_{onset} (black solid squares) is the onset of the SMP phenomenon, H_{sp} (red open circles) is the second peak position, H_{irr} (green solid triangles) is the irreversibility field, H_{c2} (blue open diamonds) is the upper critical field. The black arrow indicates the region investigated in this work. The corresponding behaviors are fitted with the expression $H(T) = H(0)\,(1-T/T^*)^n$. Fit details are reported in Ref.[10]. Inset: enlargement of the region near T_c.

Critical current density $J_c(H)$

The critical current density $J_c(H)$ have been estimated, from the original m(H) data, using the Bean critical state model[11,12]:

$$J_c = \frac{20\Delta M}{\left[a\left(1-\frac{a}{3b}\right)\right]}$$

where $\Delta M = M_{dn} - M_{up}$ is the difference between the magnetization measured for decreasing (M_{dn}) and increasing (M_{up}) applied field respectively, and a and b are the lengths (in cm) characterizing the cross section of the sample perpendicular to the applied field (H||c). The obtained $J_c(H)$ curves are reported in Figure 2 for different temperatures.

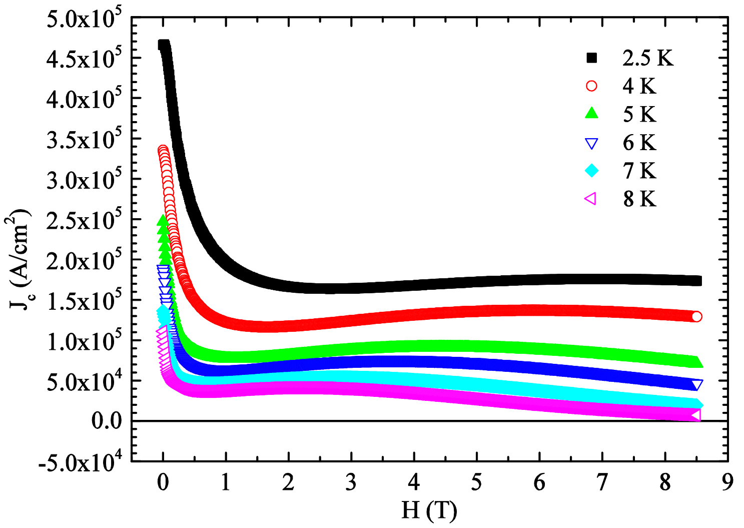

Figure 2 Critical current density J_c as a function of magnetic field at different temperatures.

Relaxation measurements M(t)

The M(t) relaxation measurements have been performed following the procedure reported in Ref.[13]. An example of time dependence of magnetization at different magnetic fields at T = 4 K has been reported in the main panel of Figure 3. All the curves have been normalized by their first value measured after reaching the target field. In the inset of Figure 3, the determination of creep rate S values has been reported. The slope of the last part of the M/M_0 vs lnt curves (about 5400 seconds after the beginning of each magnetic relaxation measurements) in order to overcome the initial exponentially fast flux motion. In this way, after so long time, all the transients in the magnetic relaxation have expired and the vortex lattice has reached its configuration which evolves just depending on the temperature and magnetic field considered.

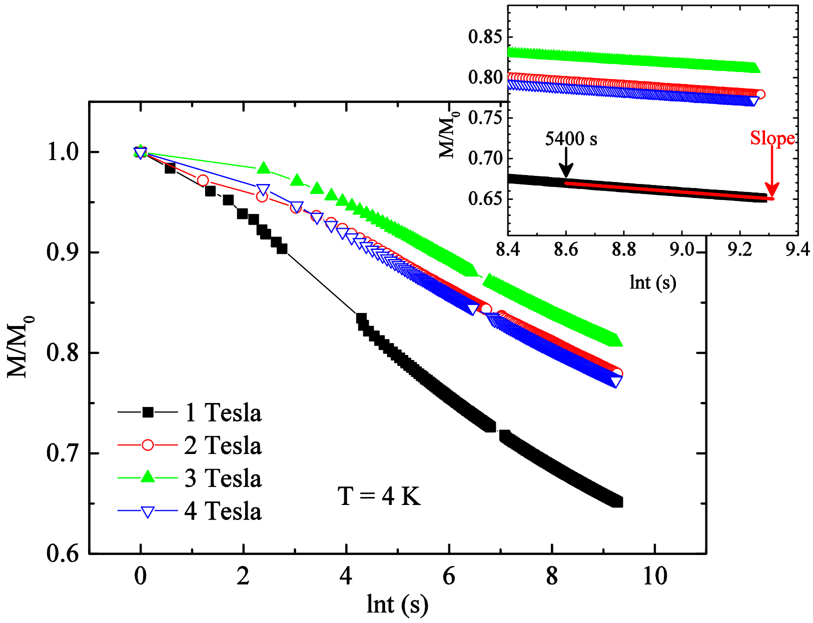

Figure 3 Time dependence of magnetization at different magnetic fields at T = 4 K. Inset: the slope of the last part of the M/M_0 vs lnt curve corresponds to the value of the creep rate S.

References

1. Zhou, W., Xing, X., Wu, W., Zhao, H. & Shi, Z. Second magnetization peak effect, vortex dynamics, and flux pinning in 112-type superconductor Ca0.8La0.2Fe1-xCoxAs2. *Sci. Rep.* **6**, 22278 (2016).

2. Salem-Sugui, S. *et al.* Flux dynamics associated with the second magnetization peak in the iron pnictide Ba1-xKxFe2As2. *Phys. Rev. B - Condens. Matter Mater. Phys.* **82**, 054513 (2010).

3. Sundar, S. *et al.* Study of the second magnetization peak and the pinning behaviour in Ba(Fe0.935Co0.065)2As2pnictide superconductor. *Supercond. Sci. Technol.* **30**, 125007 (2017).

4. Bonura, M., Giannini, E., Viennois, R. & Senatore, C. Temperature and time scaling of the peak-effect vortex configuration in FeTe0.7Se0.3. *Phys. Rev. B* **85**, 134532 (2012).

5. Shen, B. *et al.* Flux dynamics and vortex phase diagram in Ba(Fe1-xCox)2As2 single crystals revealed by magnetization and its relaxation. *Phys. Rev. B* **81**, 014503 (2010).

6. Katayama, K. *et al.* Peak effect and vortex phase diagram of YBa2Cu4O8. *Phys. C Supercond.* **392–396**, 382–385 (2003).

7. Nishizaki, T., Naito, T., Okayasu, S., Iwase, A. & Kobayashi, N. Effects of weak point disorder on the vortex matter phase diagram in untwinned YBa2Cu3Oy single crystals. *Phys. Rev. B* **61**, 3649–3654 (2000).

8. Stamopoulos, D. & Pissas, M. Hysteretic behavior of the vortex lattice at the onset of the second peak for the HgBa2CuO4+δ superconductor. *Phys. Rev. B - Condens. Matter Mater. Phys.* **65**, 1–7 (2002).

9. Stamopoulos, D., Pissas, M. & Bondarenko, A. Possible reordering of vortex matter near the end point of the second peak line in the YBa2Cu3O7−δ compound. *Phys. Rev. B - Condens. Matter Mater. Phys.* **66**, 1–7 (2002).

10. Galluzzi, A. *et al.* Evidence of pinning crossover and the role of twin boundaries in the peak effect in FeSeTe iron based superconductor. *Supercond. Sci. Technol.* **31**, 015014 (2018).

11. Bean, C. P. Magnetization of hard superconductors. *Phys. Rev. Lett.* **8**, 250–253 (1962).

12. Bean, C. P. Magnetization of High-Field Superconductors. *Rev. Mod. Phys.* **36**, 31–39 (1964).

13. Yeshurun, Y., Malozemoff, A. P. & Shaulov, A. Magnetic relaxation in high-temperature superconductors. *Rev. Mod. Phys.* **68**, 911–949 (1996).
